# Chemotherapeutic drugs sensitize human renal cell carcinoma cells to ABT-737 by a mechanism involving the Noxa-dependent inactivation of Mcl-1 or A1

**DOI:** 10.1186/1476-4598-9-164

**Published:** 2010-06-24

**Authors:** Henry Zall, Arnim Weber, Robert Besch, Niko Zantl, Georg Häcker

**Affiliations:** 1 Institute for Medical Microbiology, Technische Universität München, Trogerstr., Munich, Germany; 2 Department of Dermatology and Allergology, Ludwig-Maximilian University, Frauenlobstr., Munich, Germany; 3 Clinic of Urology, Clinic Konstanz, Luisenstr., Konstanz, Germany; 4 Institute of Medical Microbiology and Hygiene, University Freiburg, Hermann-Herder-Str., Freiburg, Germany

## Abstract

**Background:**

Human renal cell carcinoma (RCC) is very resistant to chemotherapy. ABT-737 is a novel inhibitor of anti-apoptotic proteins of the Bcl-2 family that has shown promise in various preclinical tumour models.

**Results:**

We here report a strong over-additive pro-apoptotic effect of ABT-737 and etoposide, vinblastine or paclitaxel but not 5-fluorouracil in cell lines from human RCC. ABT-737 showed very little activity as a single agent but killed RCC cells potently when anti-apoptotic Mcl-1 or, unexpectedly, A1 was targeted by RNAi. This potent augmentation required endogenous Noxa protein since RNAi directed against Noxa but not against Bim or Puma reduced apoptosis induction by the combination of ABT-737 and etoposide or vinblastine. At the level of mitochondria, etoposide-treatment had a similar sensitizing activity and allowed for ABT-737-induced release of cytochrome *c*.

**Conclusions:**

Chemotherapeutic drugs can overcome protection afforded by Mcl-1 and A1 through endogenous Noxa protein in RCC cells, and the combination of such drugs with ABT-737 may be a promising strategy in RCC. Strikingly, A1 emerged in RCC cell lines as a protein of similar importance as the well-established Mcl-1 in protection against apoptosis in these cells.

## Background

Renal cell carcinoma is the most common (85%) malignant tumour of the kidney. Although the disease can be cured by removal of the kidney in cases of localized disease, about 20% of patients have detectable metastatic disease at the time of diagnosis, and 20 - 40% of patients develop metastases following surgery. The 2 year survival of patients with metastatic disease is under 20% [1,2], reflecting the poor response of the disseminated tumour to chemo- or radiotherapy.

This resistance is at least in part the result of a low sensitivity of the tumour cells to apoptosis induction by these agents. Chemotherapeutic drugs are generally recognized as inducers of mitochondrial apoptosis, and the efficiency of this process is a determinant of the drug response [3]. Mitochondrial apoptosis is largely regulated by the Bcl-2 family of proteins [4]. This family contains both pro- and anti-apoptotic members. Apoptosis is initiated by one or several proteins from the BH3-only subgroup (eight proteins that are structurally related to each other only in their short alpha-helical BH3-domain are accepted by the majority of authors although more have been proposed), which then activate the effectors Bax/Bak. The anti-apoptotic proteins (Bcl-2, Bcl-X_L_, Bcl-w, Mcl-1 and A1) prevent this activation. Full activation of Bax or Bak results in the release of cytochrome *c *from mitochondria, the cytosolic activation of caspases and apoptosis [3]. How the activation of Bax/Bak by BH3-only proteins occurs molecularly and which members of the subgroups interact during apoptosis induction is a matter of dispute [5-7].

Anti-apoptotic Bcl-2 proteins can bind BH3-only proteins through their BH3-domains although with surprisingly strongly varying affinities [8]. This has engendered the model that anti-apoptotic proteins normally keep Bax/Bak inactive until saturated by BH3-only proteins (alone or in combination), which will allow auto-activation of Bax/Bak [6]. Others favour a model where Bax/Bak have to be activated through BH3-only proteins although this has proved difficult to show experimentally [9,10]. It is clear however that some BH3-only proteins can bind to all anti-apoptotic proteins (such as the BH3-only proteins Bim and Puma) while for instance Bad can bind only Bcl-2, Bcl-X_L_, Bcl-w but not Mcl-1 or A1. The opposite is the case for the BH3-only protein Noxa, whose binding appears to be restricted to Mcl-1 and A1 [8]. Extensive experimental evidence shows that the two anti-apoptotic groups of proteins, Bcl-2, Bcl-X_L_, Bcl-w on one hand and Mcl-1 and A1 on the other both have to be targeted to induce apoptosis [3].

Recently, feasibility of a new approach to apoptosis induction has been demonstrated in a range of tumour cells, namely the specific targeting of anti-apoptotic Bcl-2 proteins. One substance, ABT-737 has already been tested in a number of preclinical models *in vitro *and in animals and the orally better bioavailable derivative ABT-263 is at present in clinical studies [11-13]. ABT-737 binds with high affinity to the BH3-binding cleft in Bcl-2, Bcl-X_L _and Bcl-w but not Mcl-1 or A1 [11,14]. A number of malignancies show response to treatment with ABT-737 as single agent while more are sensitive to the combination of ABT-737 with other chemotherapeutic drugs (for review see [15,16]). The binding pattern of ABT-737 to anti-apoptotic proteins suggested that apoptosis resistance due to high expression of Bcl-2 would be overcome but the expression of Mcl-1 or A1 would provide protection. A number of studies have investigated this resistance to ABT-737 and have found consistently that Mcl-1 can indeed confer resistance to ABT-737 while experimental approaches that down-regulate Mcl-1 sensitize tumour cells to ABT-737 (reviewed in [16]). Since down-regulation of Mcl-1 has this strong effect, A1 seems to play no role in resistance to ABT-737 and it has been said that A1 is not expressed in most tumours although this may be a problem of sensitivity of A1 protein detection [14]. However, especially in haematological tumours a role of A1 has been found [17-19], and over-expression of A1 in mice has been described to contribute to tumorigenesis [20].

In RCC cells, easily detectable levels of Bcl-2 are expressed [21], and some association of high Bcl-2-expression with a poor prognosis in RCC has been described [22]. We have found recently that the expression of the BH3-only protein Bim was reduced in RCC [23], which may contribute to low drug sensitivity in this tumour entity. Although the binding capacity of Bim in terms of anti-apoptotic Bcl-2 proteins is broader than that of ABT-737, there is the chance that ABT-737 will nevertheless overcome apoptosis resistance of RCC when combined with other chemotherapeutic drugs, for instance by releasing the little Bim there is from its sequestration to anti-apoptotic Bcl-2 proteins. We therefore undertook this study where we tested for augmentation of ABT-737-killing by drugs in use as chemotherapeutic agents against RCC.

In cell lines *in vitro*, ABT-737 sensitized RCC cells strongly to apoptosis induction by etoposide, paclitaxel and vinblastine but not 5-fluorouracil (5-FU). In analyzing the contribution of Bcl-2 family proteins we noticed that endogenous Noxa protein was required for this sensitization, suggesting that neutralization of Mcl-1 or A1 was achieved only through Noxa. Reduction of Mcl-1 expression by RNAi rendered RCC cells sensitive to ABT-737 in the absence of additional stimuli. More surprisingly, A1-specific RNAi had a similar sensitizing effect on RCC cells. RCC cells can thus be killed efficiently if the 'Bcl' group of anti-apoptotic proteins (Bcl-2, Bcl-X_L_, Bcl-w) are targeted by ABT-737 and the group consisting of Mcl-1 and A1 by endogenous Noxa protein.

## Results

### ABT-737 enhances apoptosis induced by vinblastine, paclitaxel and etoposide but not 5-FU in RCC lines

**Figure 1:**
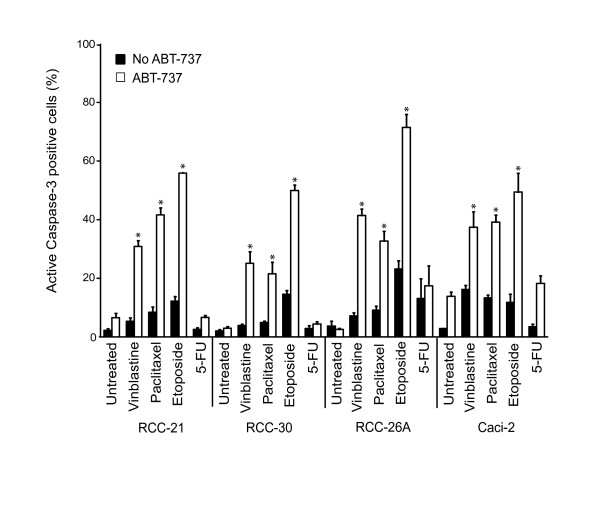
**Potent augmentation of ABT-737-killing by chemotherapeutic drugs**.Cells from clear cell RCC lines 21, 30, 26A and Caci-2 were treated with 1 μM ABT-737, 100 nM vinblastine, 200 nM paclitaxel, 200 μM etoposide, 1 mM 5-FU or with the combination of ABT-737 plus chemotherapeutic drugs. Apoptosis was quantified by staining for activated caspase-3 at 24 h. Values represent the mean/SEM of at least three independent experiments. Note that ABT-737 on its own induced significant apoptosis in the RCC cell line Caci-2. The difference between ABT-737 alone and ABT-737 + 5-FU was not statistically significant (* *P *< 0.04, single treatment versus combination treatment).

We tested four patient-derived clear cell RCC cell lines for their sensitivity to ABT-737. ABT-737 on its own was almost completely inactive. As noted previously, little apoptosis was induced by any of the chemotherapeutic drugs used. However, there was a strong, more than additive pro-apoptotic effect of ABT-737 plus three of the four other drugs tested. This effect was strongest for etoposide but still substantial for vinblastine and paclitaxel (Figure 1; Additional file 1, Figure S1A and B). No such effect was seen for the combination of 5-FU and ABT-737 in any of the lines tested, even at later time points where 5-FU induced considerable apoptosis on its own (Additional file 1, Figure S1D). No more than additive induction of apoptosis (as measured by staining for active caspase-3) or cell death (detected as PI-staining) was observed for a range of concentrations of 5-FU and ABT-737 (Figure 1; Additional file 1, Figure S1A, C and data not shown). Staining for annexin V-binding gave similar results as staining for active caspase-3 (Additional file 1, Figure S1B). Cell death induced by combination treatment was caspase-dependent as it was blocked by the caspase-inhibitor zVAD-fmk (Additional file 1, Figure S1A). ABT-737 thus can sensitize RCC cell lines for treatment with vinblastine, paclitaxel or etoposide.

### Etoposide sensitizes for ABT-737 at the level of mitochondria

**Figure 2:**
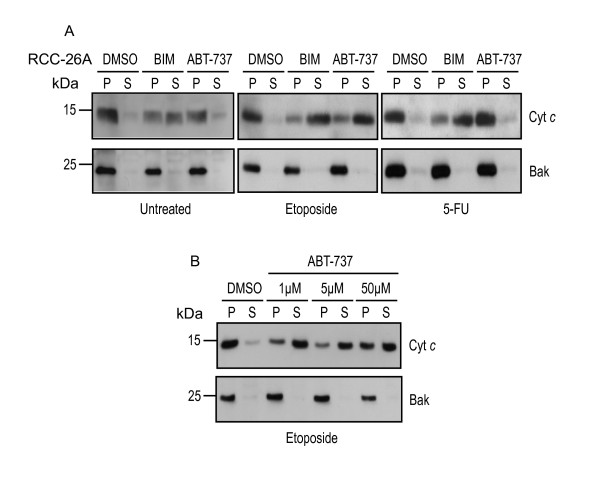
**Etoposide sensitizes RCC cells for cytochrome *c*-release by ABT-737 from mitochondria**.(**A**), (**B**), RCC-26A cells were cultured in complete medium (untreated) or treated with 200 μM etoposide or 1 mM 5-FU for 24 h. Cells were permeabilized with 200 μg/ml digitonin in the presence of solvent (DMSO, control), a peptide encompassing the Bim BH3-domain (100 μM) or ABT-737 (concentrations as shown, 50 μM in **A**). Reactions were separated into supernatants and insoluble pellets and fractions were analyzed by Western blotting for cytochrome *c*. Membranes were re-probed with a Bak-specific antibody as mitochondrial marker. P, pellet fractions, containing cytochrome *c *retained by mitochondria. S, supernatant fractions, containing released cytochrome *c*.

ABT-737 acts on Bcl-2-like proteins, which are at least predominantly localized on mitochondria. It is assumed that cytochrome *c *is released from mitochondria once all anti-apoptotic Bcl-2 family members have been neutralized [6] or when certain BH3-only proteins are liberated to activate Bax or Bak [9] (for a recent discussion see [24]), and treatment of isolated mitochondria or permeabilized cells with a peptide encompassing the Bim BH3-domain can initiate this release [5]. To obtain further evidence of the collaboration of ABT-737 and etoposide, we exposed permeabilized RCC cells that had been pre-treated with etoposide to Bim-peptide or ABT-737. As shown in Figure 2, Bim-peptide but not ABT-737 induced the release of cytochrome *c *from untreated cells from the cell line RCC-26A. This is in accordance with results in other cellular models and suggests that Bim-peptide was able to induce cytochrome *c*-release because it neutralized all Bcl-2-like proteins while ABT-737 spares Mcl-1 and A1 and therefore is inactive on its own; alternatively, the Bim-peptide may directly activate Bax or Bak. However, in cells that had been pre-treated with etoposide for 24 h and then permeabilized, ABT-737 was active in releasing cytochrome *c *(Figure 2). This suggests that etoposide-treatment had the effect of neutralizing Mcl-1 and/or A1, thereby sensitizing mitochondria for ABT-737. In line with the results obtained with intact cells, 5-FU failed to sensitize permeabilized cells to ABT-737-induced cytochrome *c*-release (Figure 2). The results therefore suggest that etoposide but not 5-FU can neutralize Mcl-1 and/or A1, leaving mitochondria sensitive to ABT-737.

### Noxa levels during treatment of RCC cells

**Figure 3:**
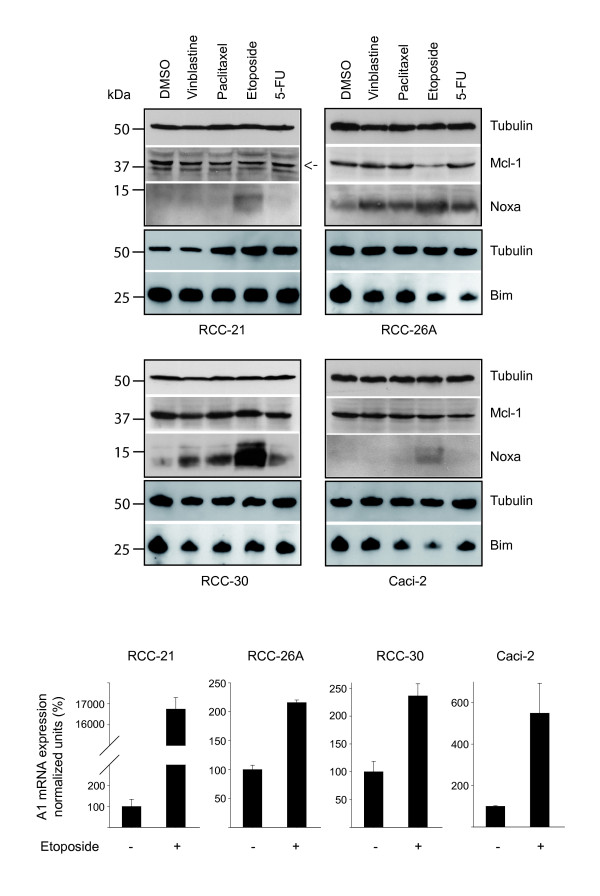
**Expression levels of Noxa, Mcl-1, Bim and A1 in RCC lines treated with chemotherapeutic drugs**.Cells from RCC cell lines 21, 26A, 30 and Caci-2 were treated for 24 h with the indicated drugs (100 nM vinblastine, 200 nM paclitaxel, 200 μM etoposide, 1 mM 5-FU). Total cell lysates were assayed for Noxa, Mcl-1 and Bim levels by Western blot analysis. Tubulin served as a loading control. The immunoblots shown here are representatives of three separate experiments. The mRNA-levels of A1 were measured by quantitative RT-PCR in cells that had been treated for 24 h with etoposide or not. The mean values of untreated controls were normalized to 100%. Data are mean/SEM from three separate experiments.

Although Mcl-1 can also bind Bim and Puma with high affinity [8], evidence for regulation of Mcl-1 activity through Noxa has been presented several times [25]. Further, etoposide-treatment seemed able to neutralize Mcl-1 and/or A1 but had only low apoptosis-inducing activity on its own, suggesting that other Bcl-2 proteins were not targeted. This indicated a role of Noxa in the treatment of RCC cells with chemotherapeutic agents since Noxa is the only BH3-only protein whose binding is limited to Mcl-1 and A1. We therefore assessed Noxa and Mcl-1 levels in RCC cell lines during treatment with these drugs. As shown in Figure 3, Noxa protein was undetectable in two and very lowly expressed in the other two cell lines used. In all cell lines, etoposide induced Noxa protein levels most strongly of the drugs tested but only in one cell line Mcl-1 was lost concomitantly (RCC-26A). In two cell lines, the other drugs failed to induce detectable levels of Noxa while in the other two all of them caused detectable induction. In these two cell lines, there was no clear difference between the drugs that potently augment ABT-737-killing (vinblastine, paclitaxel) and 5-FU, which did not have this effect. Although the results thus suggest a participation of Noxa, a number of points are not explained on the basis of these expression levels.

### Loss of expression of either Mcl-1 or A1 sensitizes RCC cells to apoptosis induced by ABT-737

**Figure 4:**
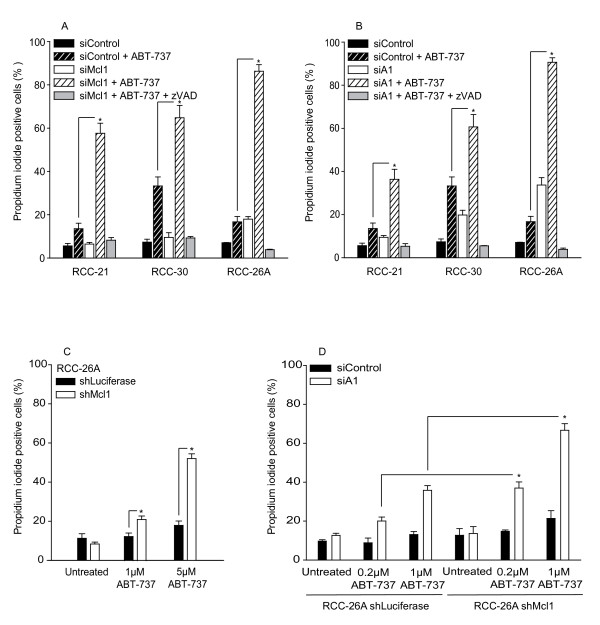
**Loss of Mcl-1 or A1 sensitizes RCC cells to apoptosis induced by ABT-737**.Cells from RCC cell lines 21, 26A and 30 were transfected with 20 nM of a control siRNA or with siRNA specific for Mcl-1 (**A**) or A1 (siRNA 441) (**B**). 48 h later, cells were treated with 5 μM ABT-737 for 24 h and cell death was measured by propidium iodide staining. In some samples 100 μM zVAD-fmk was added 1 h prior to treatment with ABT-737. (**C**), RCC cell line 26A stably expressing Luciferase (control) or Mcl-1-specific shRNA were treated with 1 μM and 5 μM ABT-737 for 24 h and cell death was measured by propidium iodide staining. (**D**), Cells from RCC-26A shLuciferase and shMcl-1 were transfected with control siRNA or A1-specific siRNA (siRNA 441). 24 h post transfection cells were treated with 0.2 μM and 1 μM ABT-737 and stained with propidium iodide 24 h later. Data are mean/SEM of three independent experiments (* *P *< 0.04, control siRNA versus Mcl-1 siRNA and A1 siRNA; shLuciferase versus shMcl-1).

As discussed above, the results suggested that etoposide and other drugs were able functionally to eliminate Mcl-1 and/or A1, enabling ABT-737 to induce apoptosis. In a number of cells it has been demonstrated that it is the expression of Mcl-1 that determines resistance to ABT-737 while A1 has been suggested not to be expressed by most tumours [14]. We decided to knock down Mcl-1 and A1 individually to test for their contributions to resistance to ABT-737. Clear although incomplete reduction of Mcl-1 protein by transfection with Mcl-1-specific siRNA was achieved in the three RCC cell lines used as well as in one cell line engineered stably to express Mcl-1-specific shRNA (Additional file 1, Figure S2A). Only very little A1 protein was detectable by Western blotting, which may be the result of low levels of expression or of low sensitivity of the available antibodies, and we failed to detect A1 protein in two of the RCC cell lines despite clear mRNA expression (Additional file 1, Figure S2B, C). However, A1 mRNA was easily detectable, and a good reduction was achieved by transfection with specific siRNA (Additional file 1, Figure S2C; in a melanoma cell line expressing detectable protein levels of A1, siRNA against A1 gave a good reduction, see Additional file 1, Figure S2B). Knock-down of Mcl-1-expression strongly sensitized RCC cells to ABT-737 (Figure 4A), adding RCC to the list of cell types where the expression levels of Mcl-1 determine susceptibility to ABT-737-induced apoptosis. Importantly, knock-down of A1 had a similar sensitizing effect (Figure 4B). There was even noticeable cell death induction by mere knock-down of A1 in the absence of additional stimuli (Figure 4B). A second siRNA directed against a separate site in the A1-mRNA had a similar sensitizing effect in the RCC cell line tested (RCC-26A, Additional file 1, Figure S3). The RCC-26A cell line stably carrying an anti-Mcl-1 shRNA construct was also sensitive to ABT-737 (Figure 4C). Additional knock-down of A1 by transient transfection with siRNA caused further sensitization for ABT-737 treatment (Figure 4D). These data indicate that resistance to ABT-737 in RCC cells is determined not only by Mcl-1 but also by expression levels of A1, and both proteins may fulfil similar functions.

### Potent augmentation of ABT-737-killing by etoposide or vinblastine requires Noxa

**Figure 5:**
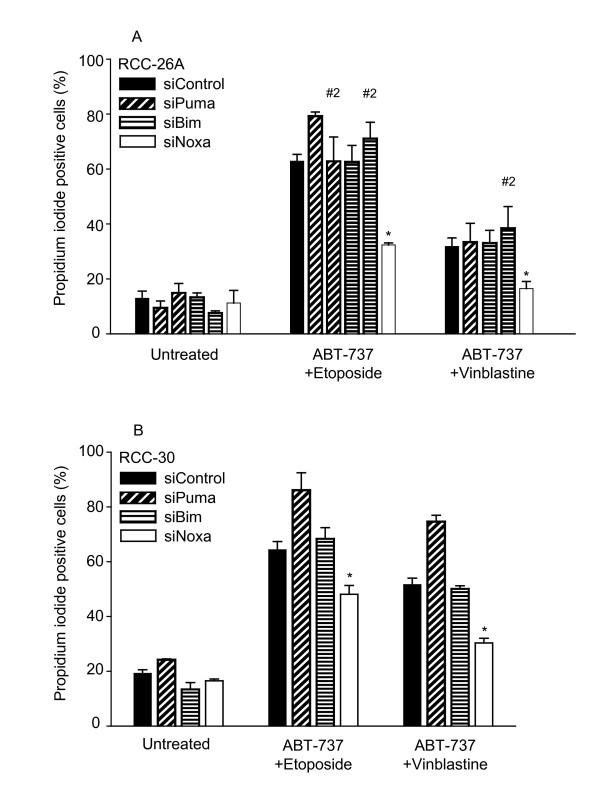
**Noxa but not Bim or Puma is required for full synergism between ABT-737 and etoposide or vinblastine**.Cells from the RCC cell line 26A (**A**) or 30 (**B**) were transfected with 20 nM of the indicated siRNAs (Bim, Puma, Noxa or control; in some experiments a second sequence targeting Bim and Puma has been included, #2). 48 h later, cells were treated with ABT-737 (1 μM) plus etoposide (200 μM) or vinblastine (100 nM) as indicated. Cell death was determined by propidium iodide staining 24 h later. Values are mean/SEM of at least three independent experiments (* *P *< 0.03; control siRNA versus Noxa siRNA).

Although the data above show an induction of Noxa upon treatment with chemotherapeutic drugs, Noxa seemed unable to cause Mcl-1 degradation in most cases, which could indicate that Noxa was not involved in apoptosis induced by combination treatments including ABT-737. Further, the BH3-only proteins Bim and Puma can also bind Mcl-1 and A1 [8] and might therefore be responsible for their neutralisation. To identify the BH3-only protein that causes this effect, we knocked down Bim, Puma and Noxa individually by transfection with specific siRNA. As shown in Additional file 1, Figure S4, the expression of the target proteins was substantially reduced upon transfection with the relevant siRNA (since basal Noxa protein levels were difficult to detect, Noxa knock-down was here done in the presence of etoposide, Additional file 1, Figure S4C). As shown in Figure 5A and 5B, no reduction of cell death was seen by the knock-down of Bim or Puma when RCC-26A or RCC-30 cells were treated with the combination of etoposide and ABT-737. However, Noxa-specific siRNA significantly reduced cell death induction by this combination. Noxa- but not Bim- or Puma-specific siRNA also inhibited cell death induced by the combination of vinblastine and ABT-737 in RCC-26A (Figure 5A) and RCC-30 (Figure 5B; for the comparative expression of Bcl-2 family proteins in RCC-26A and 30 see Additional file 1, Figure S4D). These data strongly suggest that the neutralisation of either Mcl-1 or A1 by Noxa is the effect through which chemotherapeutic drugs sensitize RCC cells to apoptosis induction by ABT-737.

These results showed the integrity of an axis where Noxa regulates the activity of Mcl-1 and A1 in RCC. Since this axis can also be used by proteasome inhibitors, we tested whether proteasome inhibition could also sensitize RCC cells to ABT-737-induced apoptosis. As shown in Additional file 1, Figure S5A, the proteasome inhibitor MG132 increased the levels of Mcl-1 and Noxa and blocked the etoposide-induced loss of Mcl-1 in RCC-26A cells. The loss of Mcl-1 during treatment with etoposide still occurred in the presence of zVAD-fmk, indicating that this loss was not due to cell death (Additional file 1, Figure S5B). MG132 further sensitized the cells for apoptosis induction by ABT-737 (Additional file 1, Figure S5C). Although etoposide induced p53-protein, the induction of Noxa by etoposide was independent of p53 (Additional file 1, Figure S5D). One possible explanation for this is that Mcl-1 (and perhaps A1) protein were stabilised but still inhibited by Noxa-binding.

## Discussion

The results of this study show that *in vitro *ABT-737-killing of RCC-cells is potently augmented by etoposide, vinblastine and paclitaxel but is surprisingly not enhanced by 5-FU. In the active combinations, the contribution of the 'traditional' chemotherapeutic drugs was the (at least in part Noxa-dependent) neutralization of Mcl-1 and/or A1 at mitochondria. Down-regulation of Mcl-1 sensitized RCC cells to ABT-737 induced apoptosis. Unexpectedly, siRNA-targeting of A1 had a very similar effect and loss of both proteins produced an additive result, suggesting that the total sum of Mcl-1 and A1 expressed in RCC cells is required to maintain viability in the presence of ABT-737.

We had previously found that the expression levels of Bim correlated with RCC-sensitivity to apoptosis, suggesting that the chemotherapeutic drugs used in part worked by activating Bim [23]. ABT-737 overcame this requirement as its pro-apoptotic activity was potently augmented by Mcl-1- or A1-knockdown. This is surprising as it suggests that Bim (in the absence of ABT-737) is activated but unable to neutralize Mcl-1, despite the high affinity of the Bim BH3-domain for Mcl-1 [8]. However, recent results in melanoma demonstrate the same effect, namely that the requirement for Bim is overcome by ABT-737 [26]. At least these relatively low levels of Bim therefore seem not to be able to antagonize the protection afforded by Mcl-1.

Although ABT-737 is active as a single agent in some cases of tumour cells, it much more commonly requires a combination partner for efficient induction of apoptosis [27,28]. It is clear that protection through high expression levels of Bcl-2 is easily overcome by ABT-737 while expression of Mcl-1 protects cells against ABT-737 [14], as does mouse A1 [20]. The main contribution of any combination partner, such as genotoxic drugs commonly used in cancer therapy, must therefore be the neutralisation of Mcl-1 and/or A1. This is clinically relevant: the results obtained in pre-clinical studies so far make it likely that the greatest success of ABT-737/ABT-263 will be in combination with chemotherapeutic drugs such as the ones in common use. However, the most potent combination partner will not necessarily be the drug that is most potent on its own but probably the one that most potently neutralizes Mcl-1 and A1. Why 5-FU was unable to cooperate with ABT-737 is unclear. 5-FU and vinblastine or paclitaxel seemed similar in their activity to induce Noxa-levels, and it would therefore be expected that they are similar in terms of sensitizing RCC cells to ABT-737. It is possible that additional mechanisms exist that control Mcl-1 and A1-inactivation consecutive to Noxa-induction but the existence of such mechanisms is completely speculative. One possibility is that Noxa is sequestered, perhaps by co-induction of an additional protein, and cannot actually bind to Mcl-1 or A1. It is further possible that 5-FU, while inducing Noxa, also increases the levels of Mcl-1/A1, perhaps by stabilizing the protein, which might counteract the pro-apoptotic effect of Noxa. More detailed studies will be required to clarify this.

In RCC, etoposide and vinblastine required endogenous Noxa for the potent augmentation of ABT-737-killing. Noxa was first described as a protein induced by phorbol-ester treatment [29]. Its function as a pro-apoptotic protein was first described as a transcriptional target of p53 [30]. Noxa can also be a transcriptional target of interferon-signalling and viral infection [31,32]. Noxa is further induced by treatment with proteasome inhibitors although this has, in melanoma, been suggested to be an indirect effect through the activation of c-myc [33]. RCC cells have usually wt p53 but p53 seems to be non-functional due to a dominant negative inhibitor [34,35]. Etoposide was found to induce p53 although the knock-down of p53 had very little effect on Noxa-induction in RCC, consistent with the concept that RCC do not have functional p53. The c-myc pathway has recently been suggested to be activated in clear cell RCC although Noxa was not identified as an up-regulated gene in that study [36]. How Noxa is activated by the drugs used here is therefore not clear.

It was surprising to note that Mcl-1 is not necessarily degraded upon treatment of RCC cell lines with agents that sensitize for ABT-737; the only situation where we observed such a decrease was treatment of one of the cell lines with etoposide. However, even in situations where Mcl-1 was not degraded Noxa was clearly involved in sensitization towards ABT-737, as shown by knock-down experiments. It has been suggested that Mcl-1 has to be degraded by the proteasome upon Noxa binding in order to be inactivated, and inhibition of the proteasome prevented the loss of Mcl-1 function [37]. This is an intriguing observation but molecularly unclear: why does Noxa when bound to Mcl-1 not suffice to neutralize its function? Moreover, there are now a number of reports showing that proteasome inhibitors can sensitize tumour cells to ABT-737, which indicates that they neutralize Mcl-1. We have found the same sensitization to ABT-737 by MG132 in our RCC cell lines in this study. Molecular details are uncertain but it seems clear on the basis of our results that Mcl-1 does not have to be degraded for the sensitization of RCC cells to ABT-737. Since targeting of A1 was also able to sensitize RCC cells, it is a possibility that the primary function of Noxa in these cases was to neutralize the function of A1 rather than Mcl-1.

A1 is a less well-investigated member of the established anti-apoptotic Bcl-2 protein group. A1 may not be expressed, at least not at high levels in many cells [14,37]. It is also possible that A1 has a very high turnover, as indeed has been suggested in a previous study. A1 mRNA was easily detectable in the cell lines we tested although we were able to detect only an uncertain signal by Western blotting. It is therefore possible that the main regulation of A1 occurs by regulating its stability. In malignant B cells, A1 has recently been described to play important roles in regulating cell survival [17,18]. As far as we know, no such role has been found in solid tumours. Intriguingly, the knock-down of either Mcl-1 or A1 was sufficient to sensitize RCC cells to ABT-737, suggesting that both proteins are necessary for survival in the presence of ABT-737. This is surprising since a distinct molecular role of Mcl-1 has been suggested, namely the sequestration of Bak. In that study, Bak was found to be sequestrated by Mcl-1 and by Bcl-X_L _while A1 was unable to fulfil this function [37] although a more recent study found that A1 could interact with and inhibit Bak [19]. Clearly, more work is required to clarify this.

In summary, both anti-apoptotic Bcl-2 proteins Mcl-1 and A1 determine the level of resistance to ABT-737 in RCC cells, and this layer of protection is disrupted by etoposide, vinblastine and probably other drugs. To understand tumour cell apoptosis in more detail and to devise rational strategies to induce apoptosis therapeutically, a better understanding of A1 function may be expected to be helpful.

## Methods

### Cell lines and materials

Human, patient-derived clear-cell renal cell carcinoma lines RCC-21, RCC-26A, RCC-30 and Caci-2 were from the German Cancer Research Centre, Heidelberg, Germany. Cells were maintained in RPMI-1640, supplemented with 10% foetal calf serum (FCS), 100 U/ml penicillin and 0.1 μg/ml streptomycin (PAA, Berlin, Germany) at 5% CO_2_, 37°C humidified atmosphere. Etoposide, paclitaxel, vinblastine and 5-fluoruracil were obtained from Sigma-Aldrich (Steinheim, Germany). ABT-737 was kindly provided by Dr. Saul Rosenberg and Dr. Steve Elmore (Abbott Laboratories, Abbott Park, NJ, USA).

### Detection of apoptosis and cell death

Cells from RCC lines were treated with the indicated drugs, harvested and washed twice in PBS, following staining with propidium iodide (5 μg/ml) in PBS or annexin V in binding buffer (10 mM Hepes pH 7.4, 140 mM NaCl and 2.5 mM CaCl_2_) and analysed within 10 min. by flow cytometry (FACS Calibur, Becton Dickinson, Franklin Lakes, NJ, USA). For detection of apoptosis, cells were fixed in 4% paraformaldehyde in PBS for 10 min. at room temperature and stained with monoclonal anti-active-caspase-3 antibody (clone C92-605, BD Pharmingen) in permeabilisation buffer (0.5% BSA and 0.75% saponin (Sigma-Aldrich) in PBS). Cells were washed in permeabilisation buffer and stained with FITC-conjugated anti-rabbit IgG secondary antibody (Dianova, Hamburg, Germany). Flow cytometric analysis was performed using a FACS Calibur (Becton Dickinson). In some experiments cells were incubated with 100 μM zVAD-fmk (Bachem, Heidelberg, Germany) 1 h prior to cell death induction.

### Immunoblotting

Cells were lysed in buffer containing 1% Triton X-100, 50 mM Tris-HCl, pH 7.4, 150 mM NaCl, 1 mM EDTA and protease inhibitor cocktail (Roche, Mannheim, Germany). Equal amounts of protein extracts were subjected to SDS-PAGE and transferred to nitrocellulose. Equal loading was confirmed by detection of tubulin using a specific antibody (clone DM 18, Sigma). Membranes were probed with antibodies directed against Bcl-2 (clone Bcl-2/100, BD Pharmingen), Bcl-X_L _(polyclonal #2762, Cell Signaling, Danvers, MA, USA), Mcl-1, cytochrome *c *(clone 22 and clone 7H8.2C12, BD Pharmingen,), Noxa (clone 114C307.1, Alexis, PA, USA,), Bim (polyclonal, B7929, Sigma), Bax, Bak (polyclonal, NT #06-499 and polyclonal, NT #06-536, Upstate, Lake Placid, NY, USA), Bcl-w (clone 31H4, #2724; Cell Signaling), Puma, Bfl-1/A1 (polyclonal #4976 and polyclonal #4647, Cell Signaling) and p53 (clone 1C12, Cell Signaling). Secondary antibodies were horseradish peroxidase conjugated anti-mouse IgG or anti-rabbit IgG antibodies (Sigma). Proteins were visualized using an enhanced chemiluminescence detection system (GE Healthcare, Buckinghamshire, UK).

### RNAi and quantitative reverse transcriptase PCR

Cells from RCC lines were transfected with 20 nM of siRNA (depicted is the 19 nt portion in the sense strand of the targeted mRNA) specific for human Mcl-1 (5'-GGUUUGGCAUAUCUAAUAA-3'), Bfl-1/A1 (441 5'-GGAAGAAUUGUAACCAU-3' and 511 5'-CGGAUGUGGAUACCUAUAA-3'), Bim (5'-GGAAGAAUUGUAACCAUAU-3' and #2 5'-CCACUAUCUCAGUGCAAUG-30), Puma (5'-CCGAGAUGGAGCCCAAUUA-3' and #2 5'-CCUGGAGGGUCCUGUACAA-3'), Noxa (5'-AGUCGAGUGUGCUACUCAA-3') or p53 (5'-GUACCACCAUCCACUACAA-3') and a control siRNA (5'-GCGCAUUCCAGCUUACGUA-3'), containing a random sequence that does not match a sequence within the human or murine genome, using Lipofectamine RNAiMAX reagent according to the manufacturer's instructions (Invitrogen, Carlsbad, CA, USA). For generation of RCC cell line 26A stably expressing Mcl-1-specific shRNA, RNAi sequences targeting Mcl-1 (5'-ACGCGGUAAUCGGACUCAA-3') and Luciferase mRNA (5'-GUGCGCUGCUGGUGCCAAC-3') were cloned in the GFP-expressing lentiviral vector pLVTHM (Dr. Didier Trono, Lausanne). Production of lentiviral particles was done by transfecting 293 FT cells (Invitrogen) together with packaging vectors pMD2.G and psPAX2 (Dr. Didier Trono, Lausanne). At 48 h post transfection with siRNA, cells were assayed for gene knockdown or were used for further experiments. Total RNA was extracted from RCC lines after siRNA knockdown using RNeasy mini kit (Qiagen, Hilden, Germany) and analysed by quantitative RT-PCR. RNA (1 μg) was reverse transcribed using Expand Reverse Transcriptase and poly (dT) oligonucleotide (Roche) according to the manufacturer's protocol. Quantitative PCR was performed using the LightCycler TaqMan Master Kit together with the Universal ProbeLibrary system (Roche). Relative gene expression is expressed as the ratio of the expression level of the gene of interest to that of hypoxanthine-phosphoribosyl-transferase (HPRT) RNA determined in the same sample.

### Cytochrome *c*-release assay

Untreated or treated cells from the RCC-26A line (2 × 10^5 ^cells per sample) were harvested and permeabilised in sample buffer (20 mM HEPES pH 7.2, 100 mM KCl, 5 mM MgCl2, 1 mM EDTA, 1 mM EGTA, 250 mM sucrose and protease inhibitor cocktail (Roche)), containing 200 μg/ml digitonin (Sigma-Aldrich). Cells were incubated for 60 min. at 30°C in the presence of BH3-only oligopeptide Bim (100 μM) or ABT-737 (1 μM, 5 μM and 50 μM). Bim-peptide (sequence MRPEIWIAQERRRIGDEFNA) was synthesized at Biosynthan GmbH (Berlin, Germany). Cells were then centrifuged for 10 min. at 13000 × g to separate them into pellet and supernatant fractions. Samples were adjusted to equivalent volumes with 4× SDS sample loading buffer and were subjected to immunoblotting.

## Abbreviations

A1: B cell leukemia/lymphoma 2 related protein A1; Bad: Bcl-2-antagonist of cell death; Bak: Bcl-2-antagonist/killer; Bax: Bcl-2 associated × protein; Bcl-2: B-cell leukemia/lymphoma 2; Bcl-X_L_: Bcl-2-like 1; Bcl-w: Bcl-2-like 2; BH3: Bcl-2 homolgy domain 3; Bim: Bcl-2 interacting mediator of cell death; BSA: bovine serum albumin; CaCl_2_: calcium chloride; CCRCC: clear cell renal cell carcinoma; c-myc: cellular myelocytomatosis viral oncogene homolog; Cyt *c*: cytochrome *c*; DMSO: dimethylsulfoxide; EDTA: ethylene diamine tetraacetic acid; EGTA: ethylene glycol tetraacetic acid; FITC: fluorescein-isothiocyanate; 5-FU: 5-fluoruracil; Hepes: 4-(2-hydroxyethyl)-1-piperazineethanesulfonic acid; IgG: immunoglobulin G; KCL: potassium chloride; MgCl_2_: magnesium chloride; Mcl-1: myeloid cell leukemia sequence 1; NaCl: sodium chloride; Noxa: (Latin for damage) phorbol-12-myristate-13-acetate-induced protein 1; p53: transformation related protein 53; PBS: phosphate-buffered saline; PI: propidium iodide; Puma: p53-upregulated modulator of apoptosis; RCC: renal cell carcinoma; RNA: ribonucleic acid; mRNA: messenger ribonucleic acid; RNAi: ribonucleic acid interference; shRNA: short hairpin ribonucleic acid; siRNA: small interfering ribonucleic acid; RT-PCR: reverse transcriptase polymerase chain reaction; SDS-PAGE: sodium dodecyl sulfate polyacrylamide gel electrophoresis; Tris: tris(hydroxymethyl)-aminomethan; Wt: wildtype; zVAD-fmk: (N-CBZ-Val-Aal-Asp(O-Me) fluoromethyl ketone.

## Competing interests

The authors declare that they have no competing interests.

## Authors' contributions

HZ contributed to conception and design, acquisition of data, analysis and interpretation of data and wrote the manuscript. AW contributed to conception, analysis and interpretation of data. RB carried out the quantitative RT-PCR and designed siRNA. NZ participated in the design of the study. GH conceived of the study, and participated in its design and coordination and wrote the manuscript. All authors read and approved the final manuscript.

## Supplementary Material

Additional file 1 **Supplementary figures 1-5**. **Figure S1 **- Potent augmentation of ABT-737-killing by chemotherapeutic drugs requires caspases. **Figure S2 **- Efficiency of the targeting of Mcl-1 or A1 by RNAi. **Figure S3 **- A1-targeting by two different siRNAs sensitizes RCC-26A cells to apoptosis induced by ABT-737. **Figure S4 **- Synergism between ABT-737 and etoposide or vinblastine requires Noxa. **Figure S5 **- MG-132 increases the levels of Mcl-1 and Noxa in the RCC-26A cell line and sensitizes for ABT-737 induced apoptosis. Click here for file
