# Supplementary material for: Chemotherapeutic drugs sensitize human renal cell carcinoma cells to ABT-737 by a mechanism involving the Noxa-dependent inactivation of Mcl-1 or A1
Source: Mol Cancer. 2010 Jun 24;9:164. doi: 10.1186/1476-4598-9-164 (PMC2901261; doi:10.1186/1476-4598-9-164)
Supplement: Additional file 1 — Supplementary figures 1-5. Figure S1 - Potent augmentation of ABT-737-killing by chemotherapeutic drugs requires caspases. Figure S2 - Efficiency of the targeting of Mcl-1 or A1 by RNAi. Figure S3 - A1-targeting by two different siRNAs sensitizes RCC-26A cells to apoptosis induced by ABT-737. Figure S4 - Synergism between ABT-737 and etoposide or vinblastine requires Noxa. Figure S5 - MG-132 increases the levels of Mcl-1 and Noxa in the RCC-26A cell line and sensitizes for ABT-737 induced apoptosis. [file 1476-4598-9-164-S1.PDF]

Additional file 1

Additional file legends

Figure S1 –Potent augmentation of ABT-737-killing by chemotherapeutic drugs requires caspases

Cells from RCC cell lines 21, 30, 26A and Caci-2 were treated with 1 µM ABT-737, 100 nM vinblastine, 200 nM paclitaxel, 200 µM etoposide, 1 mM 5-FU or with the combination of ABT-737 plus chemotherapeutic drugs. Cell death was quantified by propidium iodide (**A**) or Annexin V (**B**) staining at 24 h. (**C**) RCC cell line 26A was treated for 6 h and 12 h with ABT-737 + vinblastine or ABT-737 + etoposide and stained for activated caspase-3. (**D**) RCC cell line 26A was treated with 1 µM ABT-737, 1 mM 5-FU or with the combination of ABT-737 plus 5-FU. 100 µM zVAD-fmk was added 1 h prior to treatment with drugs in (**A**). Values are the mean/SEM of at least three separate experiments (* $P < 0.03$, single treatment versus combination treatment).

Figure S2 – Efficiency of the targeting of Mcl-1 or A1 by RNAi

(**A**), expression levels of Mcl-1 or A1 protein (**B**) in cells from RCC cell lines 21, 26A, 30, 26A shLuciferase, 26A shMcl-1 and a human melanoma cell line WM35 were analyzed by Western blotting 48 h post transfection with siRNA. Tubulin served as a loading control. As a specificity control, the levels of Mcl-1 mRNA upon A1-knock-down were analyzed (**A**). Data are means/SEM from three separate experiments. An A1 control lysate from human Burkitt lymphoma cell line Raji has been included in (**B**). Mcl-1 protein levels are shown for the line WM35 upon A1-knock-down. The immunoblots shown here are representatives of three separate experiments. **(C)**, mRNA levels of A1 in cells from RCC cell lines 21, 26A and 30

were determined by quantitative RT-PCR 48 h post transfection with siRNA. Mean values of not transfected controls were normalized to 100%. Results are means/SEM of three independent transfections.

Figure S3 – A1-targeting by two different siRNAs sensitizes RCC-26A cells to apoptosis induced by ABT-737

Cells from the RCC cell line 26A were transfected with two different siRNA sequences targeting A1 (nucleotides 511 to 530 or nucleotides 441 to 460). 48 h post transfection cells were treated with 5 µM ABT-737 for 24 h and cell death was quantified by staining for propidium iodide (**A**) or activated caspase-3 (**B**). Data obtained with siA1 441 are the same as in Figure 4 and are here reproduced for comparison. Data represents the mean/SEM of three experiments. (* $P < 0.01$, ** $P < 0.02$, control siRNA versus A1 siRNA).

Figure S4 – Synergism between ABT-737 and etoposide or vinblastine requires Noxa

Expression levels of Puma (**A**), Bim (**B**) and Noxa (**C**) protein in the RCC cell lines 26A and 30 transfected with specific or control siRNA. Cells were assayed by Western blot analysis 48 h post transfection (**A** and **B**) or were treated with 200 µM etoposide for 24 h (**C**) to induce Noxa. As a specificity control, levels of Bim and Puma were also tested upon treatment with Noxa-specific siRNA. The asterisk indicates an unspecific band. (**D**), Expression levels of Bcl-2 family proteins in the RCC cell lines 26A and 30. Tubulin served as a loading control. The immunoblots shown are representatives of three separate experiments. A1-levels were analyzed by quantitative RT-PCR (mean/SEM of three separate experiments).

Figure S5 – MG-132 increases the levels of Mcl-1 and Noxa in the RCC-26A cell line and sensitizes for ABT-737 induced apoptosis

(**A**), expression levels of Noxa and Mcl-1 were analysed by Western blotting in RCC-26A cells treated with 10 µM MG-132 and 200 µM etoposide for 16 h. (**B**), expression levels of Noxa and Mcl-1 in RCC-26A cells treated with 10 µM MG-132, 200 µM etoposide and 200 µM etoposide plus 100 µM zVAD-fmk for 6 h, 12 h and 24 h. Tubulin served as a loading control. (**C**), cells from the RCC-26A cell line were treated for 6 h and 12 h with 1 µM ABT-737 plus 10 µM MG-132 or 200 µM etoposide. Apoptosis was measured by staining for activated caspase-3. Data represent the means of three independent experiments/SEM. (**D**), cells from the RCC-26A cell line were transfected with control or p53-specific siRNA and were treated with 200 µM etoposide 24 h later. Shown are the expression levels of Noxa, Mcl-1 and p53 at 48 h post transfection with siRNA. Tubulin served as a loading control. The immunoblots shown are representatives of three independent experiments.

Figure S1

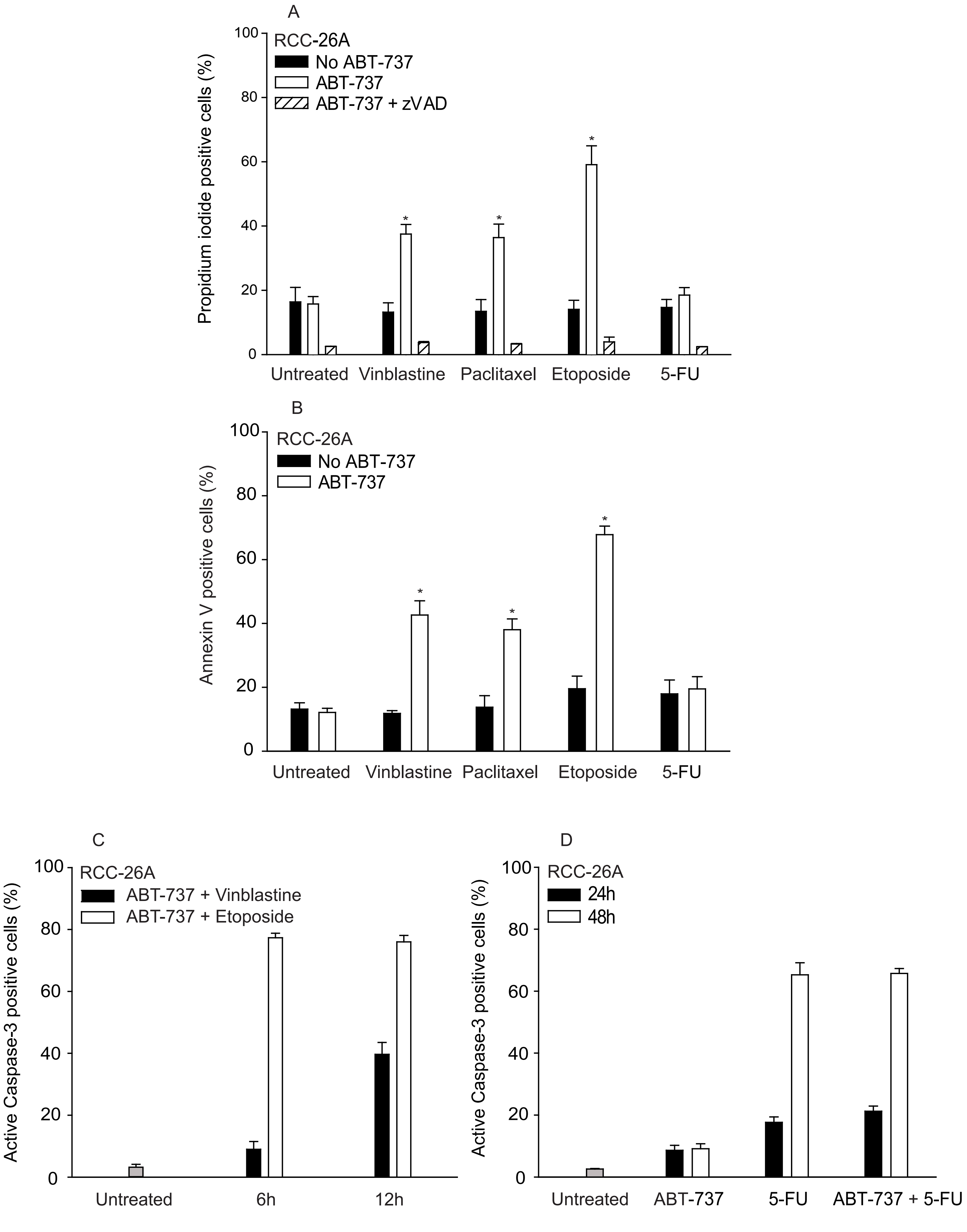
A
RCC-26A
No ABT-737
ABT-737
ABT-737 + zVAD
Propidium iodide positive cells (%)
100
80
60
40
20
0
*
Untreated
Vinblastine
Paclitaxel
Etoposide
5-FU
B
RCC-26A
No ABT-737
ABT-737
Annexin V positive cells (%)
Untreated
Vinblastine
Paclitaxel
Etoposide
5-FU
C
RCC-26A
ABT-737 + Vinblastine
ABT-737 + Etoposide
Active Caspase-3 positive cells (%)
Untreated
6h
12h
D
RCC-26A
24h
48h
Active Caspase-3 positive cells (%)
Untreated
ABT-737
5-FU
ABT-737 + 5-FU

Figure S2

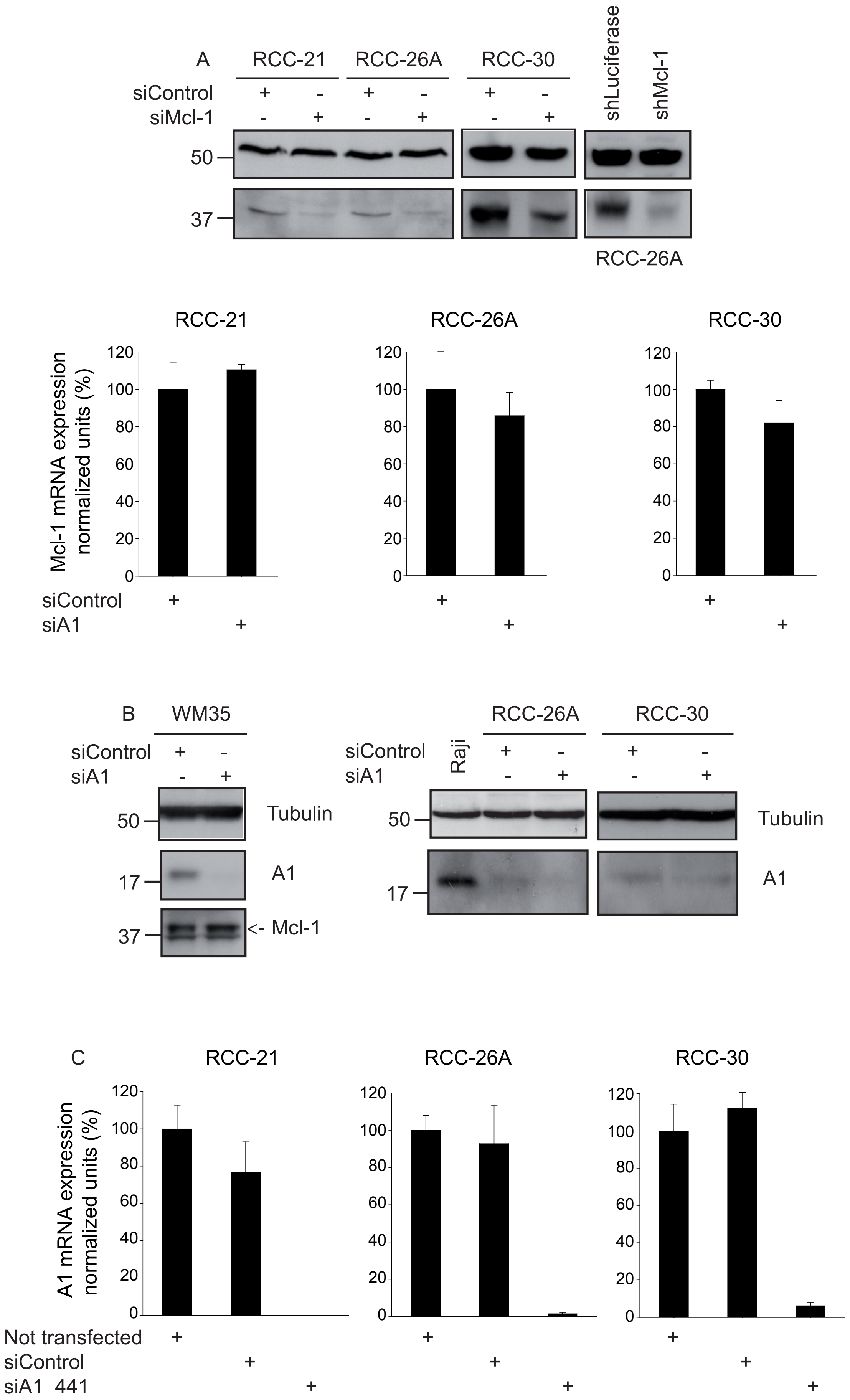
A
RCC-21
RCC-26A
RCC-30
siControl
siMcl-1
shLuciferase
shMcl-1
50
37
RCC-26A
RCC-21
RCC-26A
RCC-30
Mcl-1 mRNA expression normalized units (%)
0
20
40
60
80
100
120
siControl
siA1
B
WM35
siControl
siA1
Tubulin
A1
<- Mcl-1
50
17
37
RCC-26A
RCC-30
Raji
siControl
siA1
Tubulin
A1
50
17
C
RCC-21
RCC-26A
RCC-30
A1 mRNA expression normalized units (%)
0
20
40
60
80
100
120
Not transfected
siControl
siA1 441

Figure S3

A

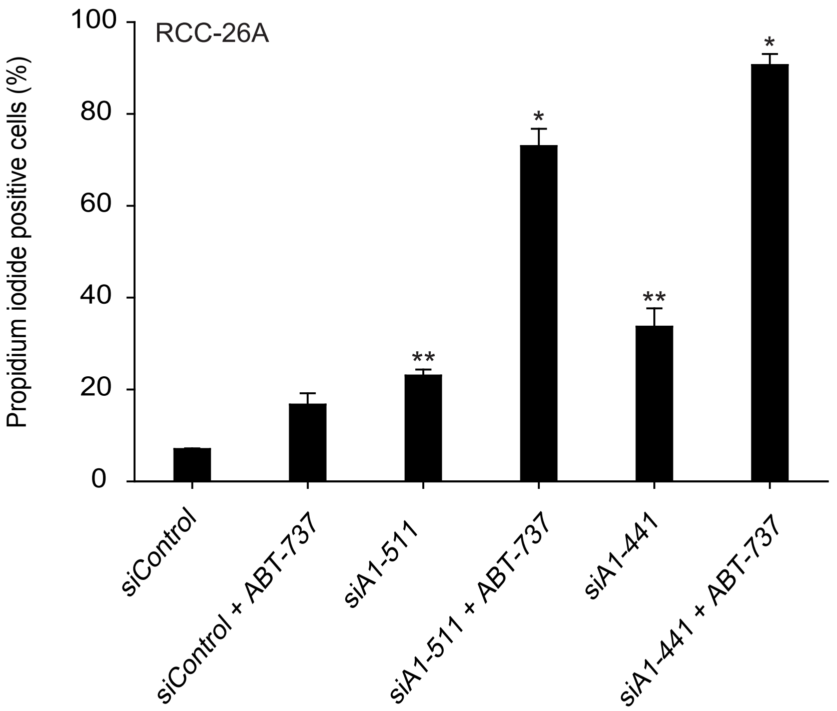
RCC-26A
Propidium iodide positive cells (%)
100
80
60
40
20
0
*
*
**
**
siControl
siControl + ABT-737
siA1-511
siA1-511 + ABT-737
siA1-441
siA1-441 + ABT-737

B

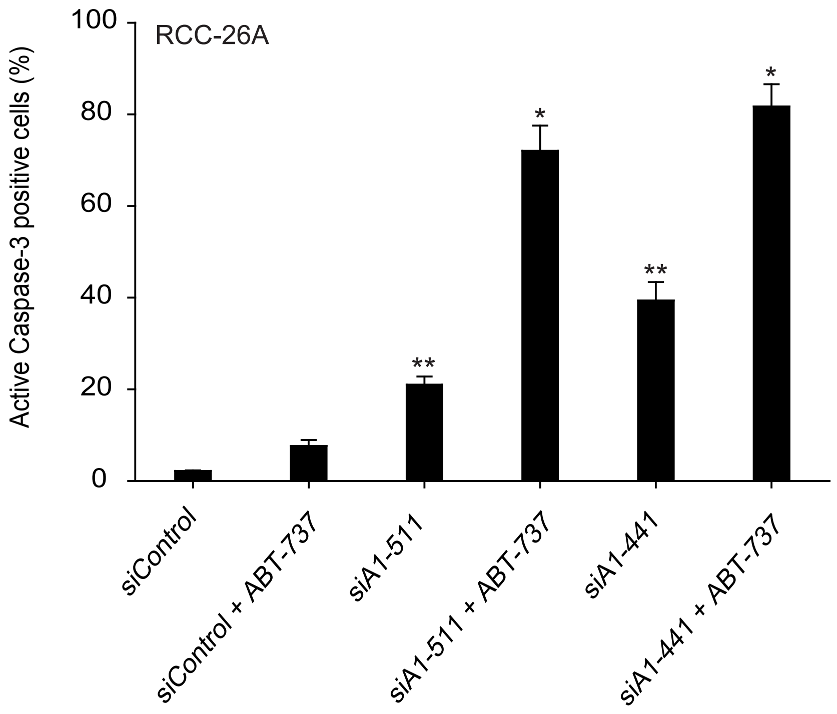
RCC-26A
Active Caspase-3 positive cells (%)
100
80
60
40
20
0
*
*
**
**
siControl
siControl + ABT-737
siA1-511
siA1-511 + ABT-737
siA1-441
siA1-441 + ABT-737

Figure S4

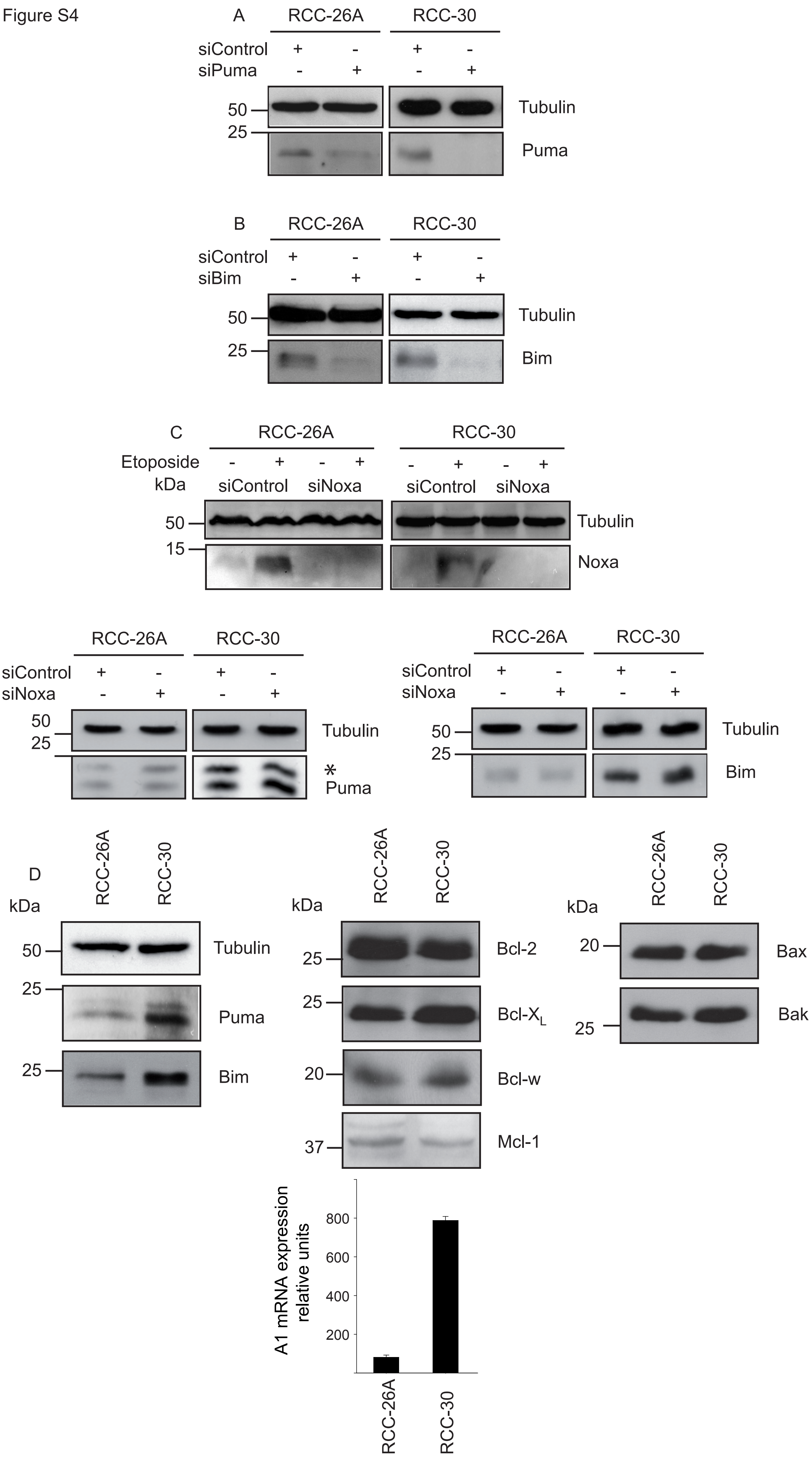

A
RCC-26A
RCC-30
siControl
siPuma
Tubulin
Puma
B
siBim
Bim
C
Etoposide
kDa
siControl
siNoxa
Noxa
*
D
Bcl-2
Bcl-X$_L$
Bcl-w
Mcl-1
Bax
Bak
A1 mRNA expression
relative units

Figure S5

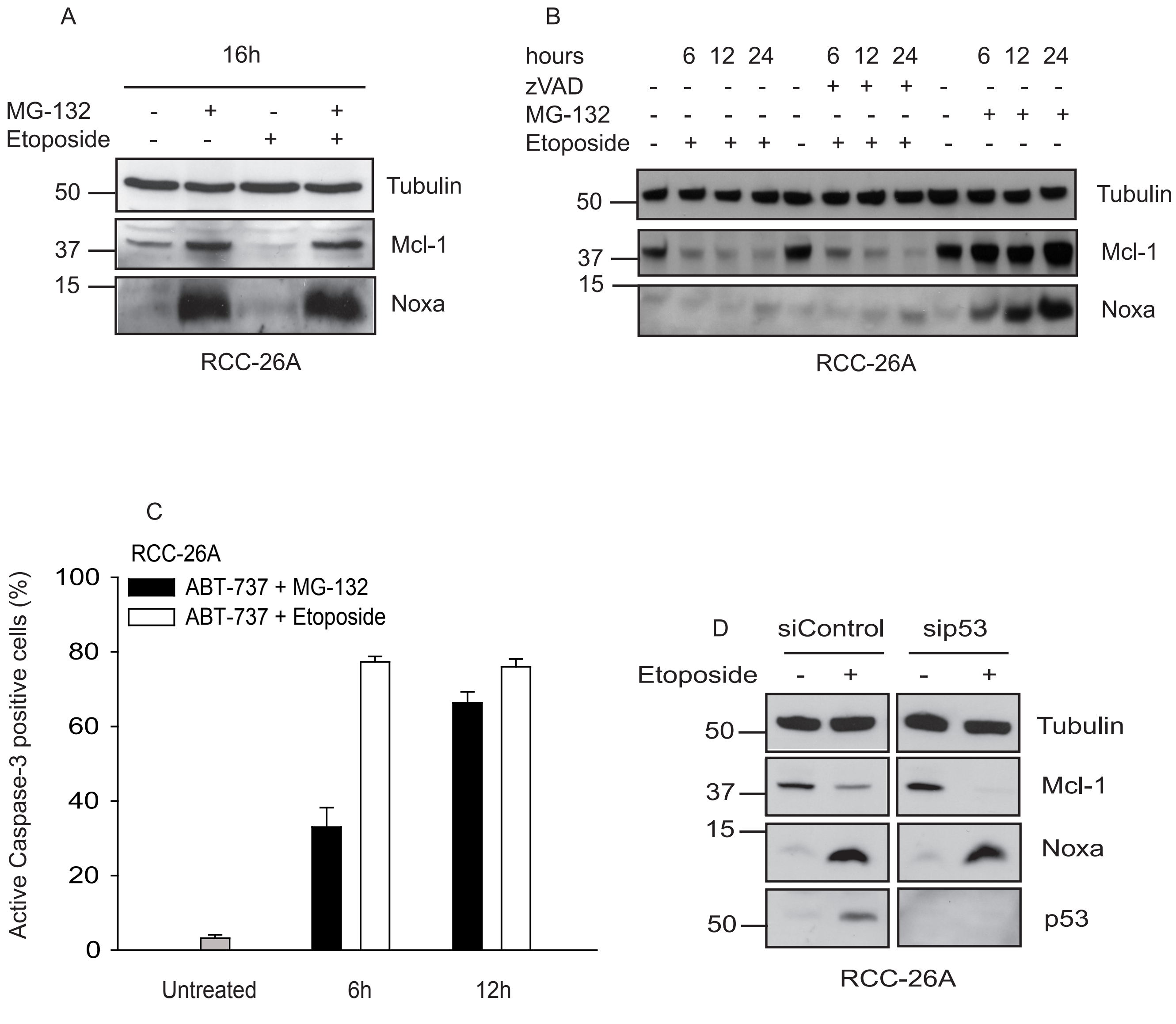

A
16h
MG-132
Etoposide
50
37
15
Tubulin
Mcl-1
Noxa
RCC-26A
B
hours
zVAD
MG-132
Etoposide
6 12 24
Tubulin
Mcl-1
Noxa
RCC-26A
C
RCC-26A
ABT-737 + MG-132
ABT-737 + Etoposide
Active Caspase-3 positive cells (%)
100
80
60
40
20
0
Untreated
6h
12h
D
siControl
sip53
Etoposide
Tubulin
Mcl-1
Noxa
p53
RCC-26A
